# Effect of apple fermentation liquid on rice growth and stress responses in saline-alkali soil under pot conditions

**DOI:** 10.3389/fpls.2026.1786329

**Published:** 2026-08-03

**Authors:** He Xu, Pan Qi, Ziyang Liu, Liquan Zhao, Jiali Zhang, Qing Wang, Bohan Jiang, Fei Huang, Sindho Wagan, Muneer Ahmed Khoso, Khuzin Dinislam, Shenglin Li

**Affiliations:** 1 School of Life Science and Agriculture Forestry, Qiqihar University, Qiqihar, Heilongjiang, China; 2 Laboratory of Pest Physiology Biochemistry and Molecular Toxicology, Department of Forest Protection, Northeast Forestry University, Harbin, China; 3 Department of Pharmacology, College of Pharmacy, Harbin Medical University, Harbin, China; 4 Department of General Chemistry, Bashkir State Medical University, Ufa, Republic of Bashkortostan, Russia

**Keywords:** apple fermentation liquid, rhizosphere fungal community, rice, saline-alkali soil, soil amelioration, sustainable agriculture, transcriptome

## Abstract

Saline-alkali soil is a major constraint in agricultural production worldwide; environmentally sustainable amendment is being adopted. This study systematically investigated the effects of different concentrations of apple fermentation liquid (AFL). On rice growth under saline-alkali soil using a controlled greenhouse pot experiment. Rice plants were treated with four AFL dilutions (1:25, 1:50, 1:100 and 1:200, v/v), and the plant growth traits, development, physiological responses, biochemical characteristics, rhizosphere fungal community composition, and root transcriptome profiles were assessed. The results showed that: (1) AFL application significantly enhanced growth of rice, with the JS25 (1:25) treatment increasing plant height, stem and root length by 70.7%, 111.4% and 72.9% respectively, compared with the saline-alkali control. It also increased the antioxidant enzyme activities CAT (114.7%-232.2%) and SOD (52.5%-119.1%), and chlorophyll content (127.1%-189.6%), while indicating alleviation of saline-alkali stress by reducing proline accumulation (53.0%-74.9%); (2) High-throughput sequencing (ITS region) revealed that AFL treatment significantly altered the structure of rhizosphere fungal community, markedly increasing the relative abundance of genera such as *Plectosphaerella*, and *Acremonium* while changes in *Fusarium* abundance were interpreted cautiously due to its taxonomic diversity; (3) Transcriptome analysis showed that AFL treatment (JS100) induced large-scale gene expression reprograming in rice roots, including genes associated with ion transport, antioxidant defense, and stress signaling pathways. Because of the post-treatment soil physiochemical parameters and qRT-PCR validation were not conducted, the proposed mechanism should be considered exploratory and hypothesis generating. Overall, this study suggests that AFL treatment may enhance rice stress tolerance under saline-alkali conditions, as reflected in the observed physiochemical, fungal, and transcriptomic responses. However, due to the absence of post-treatment soil analysis and qRT-PCR validation, these findings should be considered exploratory and hypothesis-generating.

## 1 Introduction

Saline-alkali soils characterized by high pH, excessive soluble salt content and poor nutrient availability, which adversely affects growth and poses a significant challenge on agricultural productivity and food security (Xu et al., 2023; Zhu et al., 2024). The global saline-alkali land area is over 950 million hectares of land worldwide are affected by salinization, and the total area of saline-alkali land in China is about 36 million hectares alone, and continues to expand due of improper irrigation and climate change (Gang et al., 2024). Rice (*Oryza sativa L.)*, is particular sensitive to saline-alkalinity, especially during early growth stages., Exposure to high salinity and alkalinity disrupts, it causes osmotic imbalance, ion toxicity (accumulation Na^+^), and oxidative stress, resulting in stunted growth, chlorophyll degradation, and yield degradation leads to staple for half the world’s population (Saleem et al., 2025).

The core challenge in managing saline-alkali soil lies in the simultaneous occurrence of three interrelated stress factors: high alkalinity (pH > 9-10), excessive soluble Na^+^ causing ion toxicity and osmotic imbalance, and server nutrient deficiency (particularly low N, P, and organic matter) (Zhu et al., 2024). Each of these factors requires a distinct mitigation approach. Chemical amendments such as gypsum primarily displace NA^+^ but do not correct pH or add organic matter. Physical methods like deep ploughing temporarily improve aeration but are costly and ineffective against high pH (Zhang et al., 2024). Biological approaches using slat-tolerant varieties mitigate ion uptake but do not improve soil structure of fertility (Mishra et al., 2023). Consequently, conventional single-factor strategies often fall short because they address only one component of the problem. An integrated amendment that simultaneously neutralizes alkalinity, reduces Na^+^ availability, supplies organic nutrients, and activates plant stress defenses would therefore represent a more effective and sustainable solution.

The traditional strategies for saline-alkali soil improvement include engineering approaches such as (salt leaching and deep ploughing), chemical improvement (e.g., gypsum and sulfuric acid) and biological improvement (e.g., planting salt-tolerant species and applying organic fertilizer) (Anawar and Hossain, 2023; Zhu et al., 2024). However, these methods generally suffer from high costs, long remediations cycle and or environmental risks linked with excessive chemical inputs, which leads deterioration of ecological imbalance (Yasir et al., 2025). Consequently, in recent years, improvement friendly and sustainable biologically amendment strategies getting attentions (Barbinta-Patrascu et al., 2024). AFL, generated during the fungal fermentation, a fruit derived fermentation product, it’s rich in organic acids, amino acids, vitamins and other bioactive compounds (Sadh et al., 2018; Kumari et al., 2022; Sun et al., 2022).

AFL is particularly suitable for saline-alkali soil amelioration for three interrelated reasons. (i) AFL is a strong acidic (pH 3.17-3.24), which can directly neutralize the high alkalinity (pH>10) of soda saline-alkali soils, thereby reducing Na^+^ availability and mitigating ion toxicity. (ii) AFL contains organic acids (17.56-19.97 g.L^-1^) and organic matter (2.39-2.5%) that can improve soil structure, enhance solubility of immobilized nutrients (e.g., P, K, micronutrients), and provide carbon sources for beneficial soil microorganisms. (iii) the bioactive in AFL including amino acids, vitamins, and plant growth regulators may directly stimulate plant stress-response pathways, as evidenced by increased antioxidant enzyme activity and reduced proline accumulation in this study. Unlike chemical amendments such as gypsum or sulfuric acid, AFL is derived from agricultural waste (apple drop fruits), making it cost-effective, environmentally friendly, and aligned with circular economy principles. These multifunctional properties position AFL as a promising integrated solution for saline-alkali soil improvement.

Studies have shown that fruit and vegetable AFL can effectively adjust soil pH value, improve soil physical and chemical properties, enhance soil enzyme activity and promote fungal reproduction (Rathore et al., 2023; Abidin et al., 2024). In addition, the bioactive substances in the fermentation broth can activate the plant stress-resistance defense system, improve the saline-alkali resistance of crops, and promote nutrient absorption and biomass accumulation (Ma et al., 2025). However, at present, the research on the fungal mechanism of improving saline-alkali soil by fruit and vegetable enzyme fermentation broth and its comprehensive impact on crop growth is still relatively scarce, which limits the popularization and application of this approach (Li et al., 2025; Ma, 2025). Despite these potential benefits, AFL influences plant performance under saline-alkali stress remains insufficiently understood. In particular, the relative contribution of chemical effects (acidification), shifts of fungal community, and the plant molecular responses have not been clearly revealed. Rhizosphere fungal community play a key function in mediating plant adaptation to abiotic stress via regulating nutrient cycle, production of phytohormones, and signaling pathways related to stress (Lu et al., 2021; Li et al., 2024b; Luo et al., 2020). Fungal communities, specifically those contributing to organic matter decomposition and nutrient transformation; though, the genus level functional interpretation should be approached carefully due to high taxonomic and ecological diversity (Zanne et al., 2020; Zhang et al., 2023).

Therefore, the central problem addressed by this study whether AFL, as a multifunctional biological amendment, can simultaneously alleviate the multiple stressors of saline-alkali soil (high pH, Na+ toxicity, nutrient deficiency), to promote rice growth. The proposed solution AFL application is hypothesized to be suitable because: (i) its acid nature directly counters soil alkalinity; (ii) its organic constituents enhance soil fertility and microbial activity; and (iii) its bioactive compounds activate plant stress responses. To test this hypothesis, we conducted a controlled greenhouse pot experiment examining rice growth, physiological responses, rhizosphere fungal community shifts, and root transcriptomic changes following AFL treatment at different concentrations.

## 2 Materials and methods

### 2.1 Experimental materials

#### 2.1.1 Preparation of AFL

AFL was prepared using healthy disease-free high-quality apple drop fruits (primarily the Hanfu variety) selected as fermentation substrate. Apples were washed thoroughly, cored, and cut into 1–2 cm pieces, and fermentation broth was prepared according to mass ratio of brown sugar: apple: deionized water of 1:3:10. After thoroughly mixing, with the apple-sugar, mixture was allowed to stand for 2 h prior to water addition. The fermentation material was used edible brown sugar served as the fermentation adjuvant, and deionized water was used as the fermentation medium. Then the mixture was transferred into a 5-L food-grade polyethylene plastic container, filled to no more than 80% capacity of the container volume. The opening of container was sealed with four layers of sterile gauze to allow gaseous exchange and incubated at room temperature (25 ± 2°C) in darkness. Fermentation period was 90–100 days with daily manual agitation. The fermentation was considered complete when the pH value stabilized between at (3.17 -3.24) and the electrical conductivity fluctuations remained below 5% for one week. The fermented mixture was filtered through four layers of gauze to obtain the supernatant, which was stored in sealed brown glass bottles at 4°C for subsequent use.

#### 2.1.2 Saline-alkali soil used in the experiment

Saline-alkali soil was collected from the Ailianbao Garden, Longfeng District, Daqing City, Heilongjiang Province (46°36’ N, 125°08’ E). The soil represents typical soda saline-alkali soil, with an initial pH of 10.08 ± 0.02. Control soil was collected from Boyi Ecological Park (47°11’ N, 124°52’ E) in Lindian County, Daqing City, Heilongjiang Province, with a pH of 7.88 ± 0.02. After collection, visible plant residues and stones were removed. The soils were then naturally air-dried, ground and passed by 2 mm sieve, and samples were stored in self-sealing bags for subsequent use. Pots-treatment soil physicochemical properties were not directly measured in this study; therefore, soil modification effects are inferred indirectly.

#### 2.1.3 Rice varieties and seed preparation

The conventional japonica rice (Oryza sativa L.) cultivar “Qijing No.7” was selected as the test material. The seeds were surfaced-disinfected with 75% alcohol for 5 min, washed with sterile water for three times, and incubated under controlled conditions to accelerate germination.

### 2.2 Experimental methods

#### 2.2.1 Pot experiment

Totally randomized design was adopted, and five treatments were set: CK (control, deionized water), JS200 (AFL solution diluted 1:200, v/v), JS100 (1:100), JS50 (1:50) and JS25(1:25). Each treatment included three biological replicates, with a total of 15 pots. The experimental plastic pots (upper diameter 27.5 cm, lower diameter 20 cm and height 25 cm), filled with5 kg of saline-alkali soil (dry soil weight). The pot experiment employed a completely randomized design (CRD) with five treatments and three biological replicates per treatment, totaling 15 pots. Pots were arranged randomly on greenhouse benches and repositioned weekly to minimize positional effects. The greenhouse maintained a 14 h light/10 h dark photoperiod, light intensity of 600-800 µmol.m-2. s-1 (supplemental LED lighting when needed), relative humidity of 60-70%, and temperature at 25-30 °C (day)/18-22 °C (night). No blocking factor was applied.

Rice seedlings were transplanted in a seedling tray of 32.5 cm×24.5 cm×4.5 cm for stage 40 d (of three-leaves-one-heart), and healthy seedlings with consistent growth were selected and transplanted into pots, and 5 plants were planted in each pot, arranged in a plum blossom shape with a spacing of about 8 cm. During the experiment, flooding management was adopted to keep the depth of water layer 2–3 cm.

AFL was applied using combined “base application + topdressing” approach. At transplanting 200 mL of the corresponding AFL solution mixed with soil evenly. Subsequently, 100 mL of AFL, was applied after every 7 days for total 10 applications. while the control group was applied with the equal amount of deionized water. No pH-adjusted or organic-acid control treatment was included; therefore, the relative contribution of AFL bioactivity and soil acidification could not be independently evaluated. No pH-adjusted or organic-acid control treatment was included; therefore, the relative contribution of AFL bioactivity and soil acidification could not be independently evaluated. The experiments were conducted in a greenhouse maintained at 25-30 °C under natural light conditions. Pots were rotated every three days to minimize positional effects, and flooding irrigation was maintained at a water depth of 2–3 cm.

#### 2.2.2 Sample collection

At the 70th day (maturity) after transplanting, three plants were randomly selected for each treatment to determine the growth and physiological analysis. At the same time, the rhizosphere soil samples were collected using shaking off method: the loosely attached soil was removed, and the soil tightly attached to the root surface (<2 mm away from the root surface) was collected, (~50 g) per pot. The rhizosphere soil sample is divided into two parts after removing the visible root residue: one part is naturally air-dried and screened by 2 mm sieve for soil physical and chemical properties analysis; the other part was immediately put into a sterile freezing tube, quickly frozen in liquid nitrogen and stored at -80°C for transcriptomic analysis. Rice root samples were washed with sterile deionized water, blotted dry, ~1 g of young root tissue (0–3 cm from the root tip) were selected, and immediately frozen in liquid nitrogen and stored at -80°C for transcriptomics analysis. All sample collection processes are carried out on ice.

### 2.3 Determination indicators and methods

#### 2.3.1 Physical and chemical indices of apple fermentation liquid

The AFL pH value was measured using a Leimagnetic PHS-3C pH meter. The electrical conductivity was measured using the electromagnetic DDS-307 conductivity meter. The total acid content was determined by sodium hydroxide (NaOH), titration, and calculated as citric acid equivalents. The content of organic matter was determined using potassium dichromate oxidation method. All indicators were determined in triplicates.

#### 2.3.2 Rice growth and physiological measurements

Chlorophyll content was determined by ethanol extraction: 0.1 g fresh leaf tissue was ground in 10 mL of 95% ethanol, incubated in darkness for 24 h, and absorbance measured at 665 nm, 649 nm, and 470 nm using UV/VIS spectrophotometer Model 723PC (Jinghua Instruments, Shanghai, China). Total chlorophyll was calculated as described by (Lichtenthaler, 1987). Proline content was measured using the ninhydrin method: 0.1 g fresh leaf tissue was homogenized at 100 °C for 60 min. After cooling, toluene was added, and absorbance at 520 nm was measured. Catalase (CAT) activity was assayed by monitoring H2O2 decomposition at 240 nm for 3 min, and superoxide dismutase (SOD) activity was determined using the nitroblue tetrazolium (NBT) photochemical reduction method, measuring inhibition of NBT reduction at 560 nm. While Malondialdehyde (MDA) content was measured using the thiobarbituric acid (TBA) method, with absorbance read at 532 nm and corrected for non-specific absorption at 600 nm. All assays were performed in triplicate for each biological replicate.

#### 2.3.3 Rhizosphere fungal community analysis

The total RNA extracted from the rhizosphere soil using the FastDNA^®^ SPIN Kit for Soil kit (MP Biomedicals, USA). The concentration and purity of extracted DNA were detected using NanoDrop 2000 spectrophotometer (Thermo Fisher Scientific, USA), and the integrity was detected by 1% agarose gel electrophoresis. Qualified samples were stored at -20°C for later use.

The ITS1 region of fungi was amplified by PCR with primers ITS1F (5’-cttggtcatttagagaggaagtaa-3’) and ITS2(5’-GCTGCGTTCTTCATCGATGC-3’). The PCR reaction system (25 μL) includes 2×Taq Master Mix 12.5 μL, primers (10 μmol/L) 1 μL each, DNA template 2 μL(50 ng) and ddH_2_O 8.5 μL l. The PCR amplification procedure is: pre-denaturing at 95°C for 3 min;; 30 cycles of denaturation at 95°C for 30 s, annealing at 55°C for 30 s, and extension at 72°C for 45 s; 72°C for 10 min. Each sample has 3 technical repetitions.

The PCR products were purified, quantified, and sequenced on Illumina NovaSeq 6000 platform (Illumina, USA) (PE250). Bioinformatics analysis was conducted using QIIME2 software (version 2020.2) with Operational Classification Unit (OTU) under 97% similarity threshold and taxonomic annotation performed against the UNITE database (v8.3). Functional roles of the fungal taxa were inferred at the genus level and interpreted cautiously due to limitations of ITS-based classification.

#### 2.3.4 Root transcriptome sequencing and analysis

Total RNA extracted from rice root tissues using TRIzol reagent and RNA integrity was assessed using an Agilent 2100 bioanalyzer. The libraries were constructed following Illumina protocols and sequenced on the NovaSeq 6000 platform with a reading length of 150 bp paired-end.

Clean reads were mapped to the rice reference genome, and the gene expression levels were calculated using FPKM method. The screening criteria of differentially expressed genes were identified using | logFC |≥ 1 and FDR<0.05. GO function enrichment analysis and KEGG pathway enrichment analysis were conducted to identify stress related- pathways. The RNA-seq data were not validated by q-RTPCR and are therefore presented as exploratory.

## 3 Statistical analysis

All data are presented as mean ± standard error (SE). Statistical analyses were performed using SPSS 26.0, and One-way ANOVA followed Ducan’s multiple range test was used to assess differences among treatments (P<0.05). Pearson correlation analysis was used for correlation analysis. The α diversity of fungal communities were evaluated using Chao1, Shannon and Simpson indices, and the β-diversity was visualized by principal coordinate analysis (PCoA) and non-metric multidimensional scaling analysis (NMDS). Transcriptome data analysis was carried out by R language related software package, and cluster analysis and heat map drawing of differentially expressed genes were completed by heatmap package.

## 4 Results and analysis

### 4.1 Basic characteristics of AFL

After 90–100 days, AFL applied is strongly acidic, with pH 3.17-3.24, which accords with the typical characteristics of biological fermentation products of fruits and vegetables. The conductivity is 28.34-31.47 mS·cm⁻¹, the total acid content is 17.56-19.97 g·L⁻¹, and the organic matter content is 2.39%-2.57%. The difference of physical and chemical indexes between the two batches of products is small, which indicates that the preparation process is stable and reliable (**Table 1**).

**Table 1:** Basic characteristics of apple fermentation liquid.

Batch	pH value	Conductivity/(mS/cm)	Total acid content/(gl)	Organic matter content/%
1	3.17 ± 0.02	31.47 ± 0.57	17.56 ± 1.47	2.57 ± 0.09
2	3.24 ± 0.04	28.34 ± 0.49	19.97 ± 1.04	2.39 ± 0.04

### 4.2 Chemical properties of test soils

The analysis of chemical properties of the tested soils shows (**Table 2**), that there are significant differences in physical and chemical properties between saline-alkali soil and ordinary soil. The pH value of saline-alkali soil is 10.08 0.02, which belongs to strong alkaline soil. In terms of nutrient elements, the contents of total nitrogen, total phosphorus and total potassium in saline-alkali soil are 0.52 0.02, 0.35 0.02 and 9.16 0.32 g kg, respectively, which are only 15.0%, 53.8% and 70.6% of those in ordinary soil. There is a serious shortage of available nutrients, and the contents of available nitrogen, available phosphorus and available potassium are 33.55 1.84, 25.06 1.65 and 162.62 0.41 mg kg, respectively, which are 7.4%, 18.3% and 17.6% of that of ordinary soil. The content of organic matter is only 7.12 0.25 g kg, which is much lower than that of ordinary soil (52.45 0.40 g kg). These data show that the saline-alkali soil tested has typical characteristics of high pH value, low fertility and poor nutrients.

**Table 2:** Basic chemical properties of tested soils.

Chemistry	Common planting soil	Saline soil
pH	7.88 ± 0.02^a^	10.08 ± 0.02^b^
Total N (g.kg^-1^)	3.46 ± 0.05^a^	0.52 ± 0.02^b^
Total P/(g.kg^-1^)	0.65 ± 0.02^a^	0.35 ± 0.02^b^
Total K/(g.kg^-1^)	12.98 ± 0.14^a^	9.16 ± 0.32^b^
Alkaline hydrolyzable N/(mg.kg^-1^)	451.81 ± 2.84^a^	33.55 ± 1.84^b^
Available P/(mg.kg^-1^)	136.79 ± 5.43^a^	25.06 ± 1.65^b^
Available K/(mg.kg^-1^)	923.53 ± 1.27^a^	162.62 ± 0.41^b^
Organic matter/(g.kg^-1^)	52.45 ± 0.40^a^	7.12 ± 0.25^b^

Different lowercase letters after peer data indicate significant differences (P<0.05).

### 4.3 Effect of AFL on growth and development of rice

#### 4.3.1 Morphological indicators of rice

**Figure 1:**
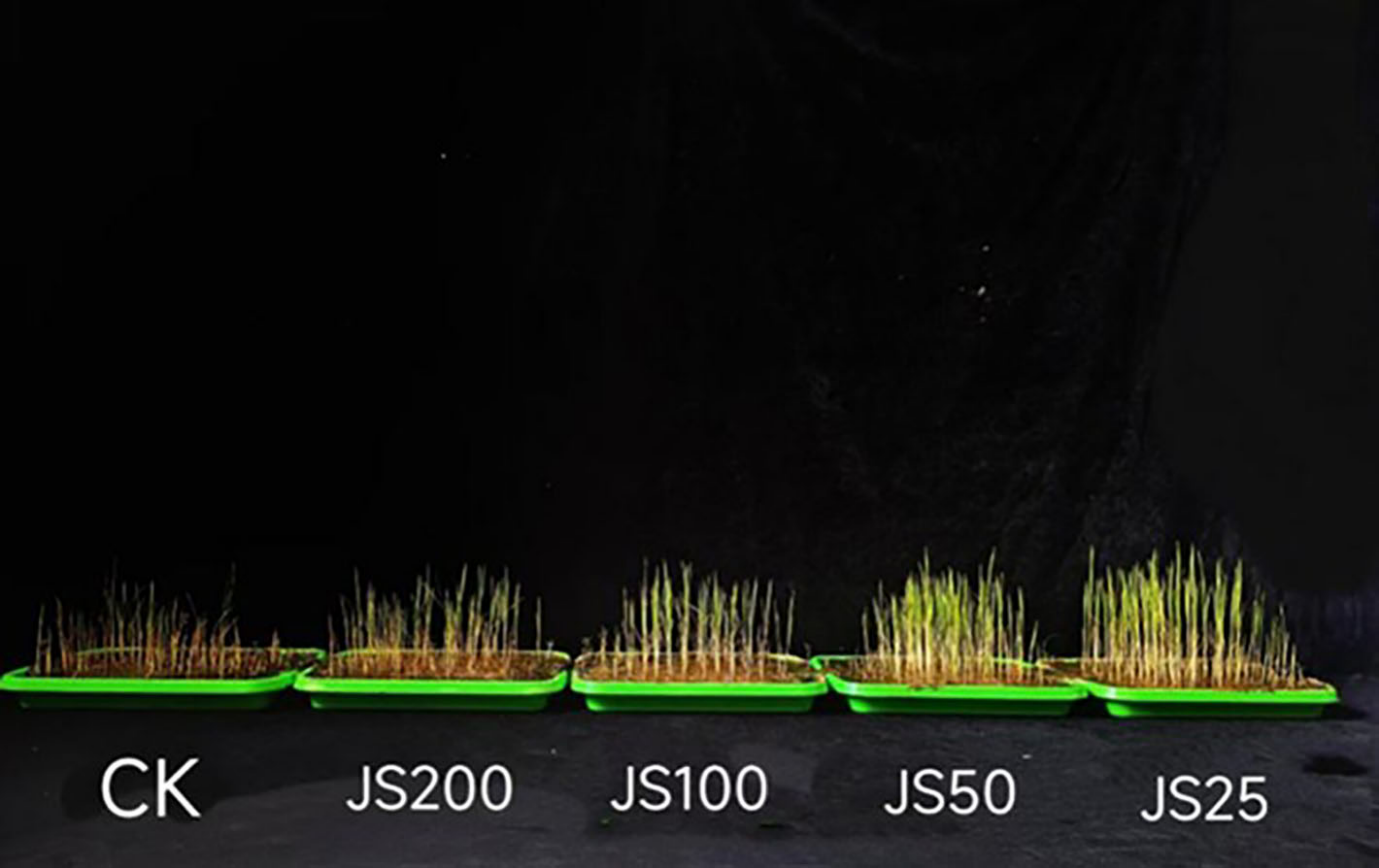
Effects of AFL on rice seedling growth under saline-alkali stress. CK: control (deionized water). JS25, JS50, JS100, JS200: AFL dilutions of 1:25, 1:50, 1:100, and 1:200 (v/v), respectively. JS25 treatment produced the tallest and most robust seedlings with dark green leaves, while CK showed severe growth inhibition and leaf yellowing.

From the overall growth of rice seedlings (**Figure 1**), the effects of different concentrations of AFL on promoting rice growth varied significantly. The control group (CK) showed severely inhibited growth, with stunted plants, fewer tillers, and yellowing leaves, exhibiting typical symptoms of salt-alkali stress. As the concentration of the fermentation broth increased (dilution factor decreased), the rice growth gradually improved. Rice seedlings treated with JS200 showed slight improvement compared to the control, but their overall growth remained weak. Rice seedlings treated with JS100 and JS50 showed significant improvement, with increased plant height, greener leaves, and more tillers. The JS25 treatment was the most effective, producing the most vigorous seedlings with tall, robust plants, dark green leaves, and the highest number of tillers; overall growth was similar to that of seedlings grown in normal soil.

##### 4.3.1.1 Plant height change

JS25 treatment had the most significant effect, with the plant height of 21.47 ± 0.76 cm, which was significantly higher than that of the control (12.58 0.84 cm) (P < 0.001). The plant height of JS50 and JS100 treatments were 19.32 ± 0.63 cm and 18.41 ± 0.72 cm, respectively, which were also significantly higher than the control (P<0.001). The plant height of JS200 was 16.94 ± 0.59 cm, which was still significantly higher than that of the control (P<0.001). The plant height of each treatment was JS25>JS50>JS100>JS200>CK, which showed a trend of gradually increasing with the increase of yeast concentration (dilution multiple decreased) (**Figure 1A**).

##### 4.3.1.2 Variation of stem length

**Figure 2:**
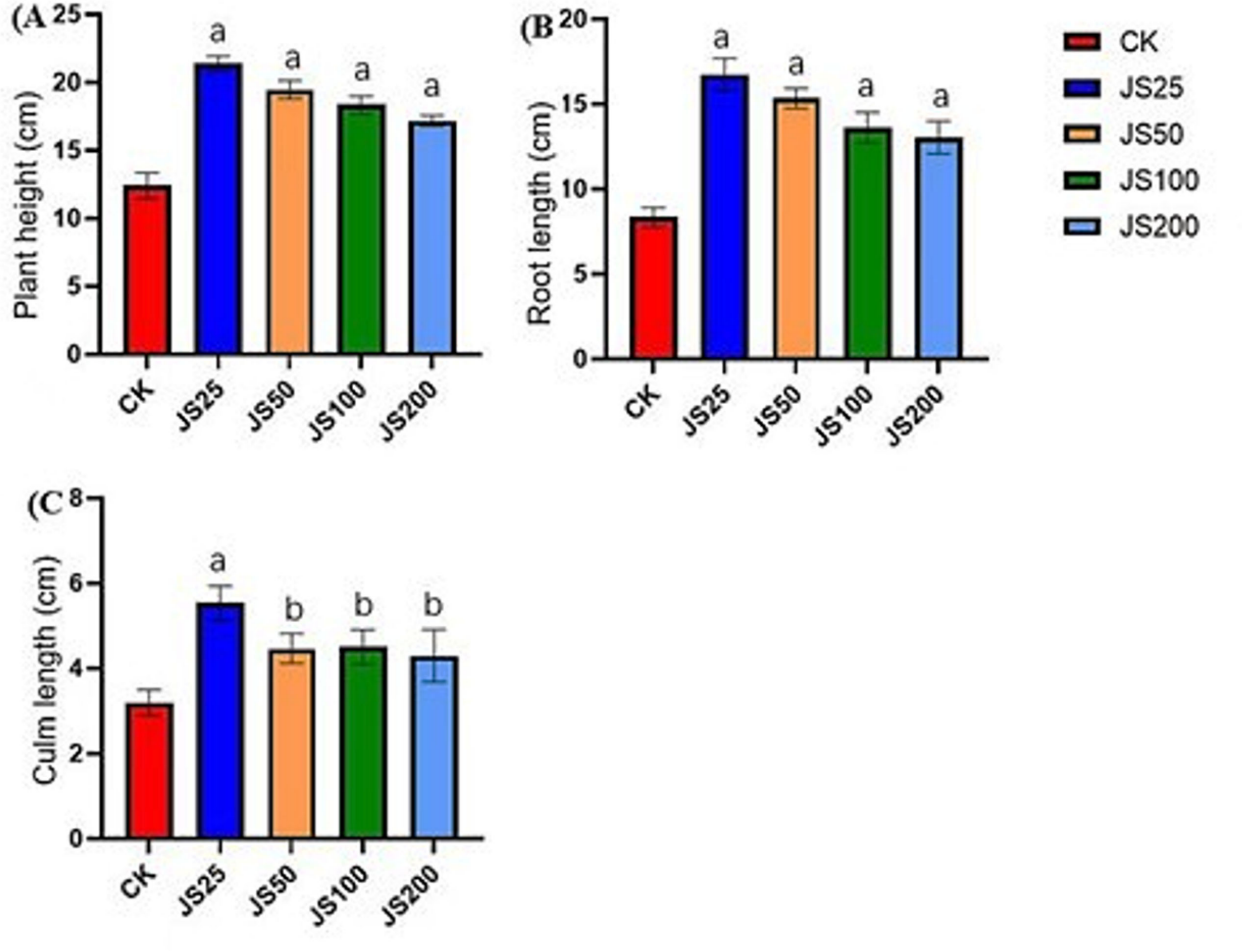
Effects of AFL treatment on rice plant morphology. **(A)** Plant height; **(B)** Stem length; **(C)** Culm length. *** indicates extremely significant difference compared to the control group (CK) (P<0.001), ** indicates significant difference (P<0.01). CK: Control (deionized water); JS25, JS50, JS100, and JS200 represent 1:25, 1:50, 1:100, and 1:200 (v/v) dilutions of AFL, respectively. Data are mean ± standard error (n=3).

The variation law of stem length is basically consistent with plant height. The stem length of JS25 treatment was 16.83 ± 0.91 cm, which was significantly higher than that of the control (7.96 0.67 cm, P < 0.001), and it was the best among all treatments. The stem lengths of JS50, JS100 and JS200 treatments were 15.14 ± 0.58 cm, 13.26 ± 0.74 cm and 12.43 ± 0.83 cm, respectively, which were significantly higher than the control (P<0.001). The promotion effect of stem length is more obvious than that of plant height, which shows that AFL mainly regulates plant height by promoting stem elongation, which is consistent with the mechanism of organic acids and growth regulators in fermentation broth promoting cell elongation and growth (**Figure 2A**).

##### 4.3.1.3 Culm length changes

AFL treatment significantly promoted the elongation of rice main roots. The root length of JS25 treatment was 5.48 ± 0.37 cm, which was significantly higher than that of control (3.17 0.29 cm, P < 0.001). The root lengths of JS50, JS100 and JS200 treatments were 4.36 0.41cm, 4.52 0.33cm and 4.19 0.46cm, respectively, which were significantly higher than the control (P<0.01). The root length of each treatment is JS25>JS100>JS50>JS200>CK (**Figure 2A**).

Different concentrations of AFL treatment significantly affected the plant height, stem length, and root length of rice cultivated in saline-alkali soil (**Figure 2**). All treatments significantly promoted rice growth and development, and showed a certain concentration-dependent effect.

#### 4.3.2 Rice biomass

##### 4.3.2.1 Total fresh weight

**Figure 3:**
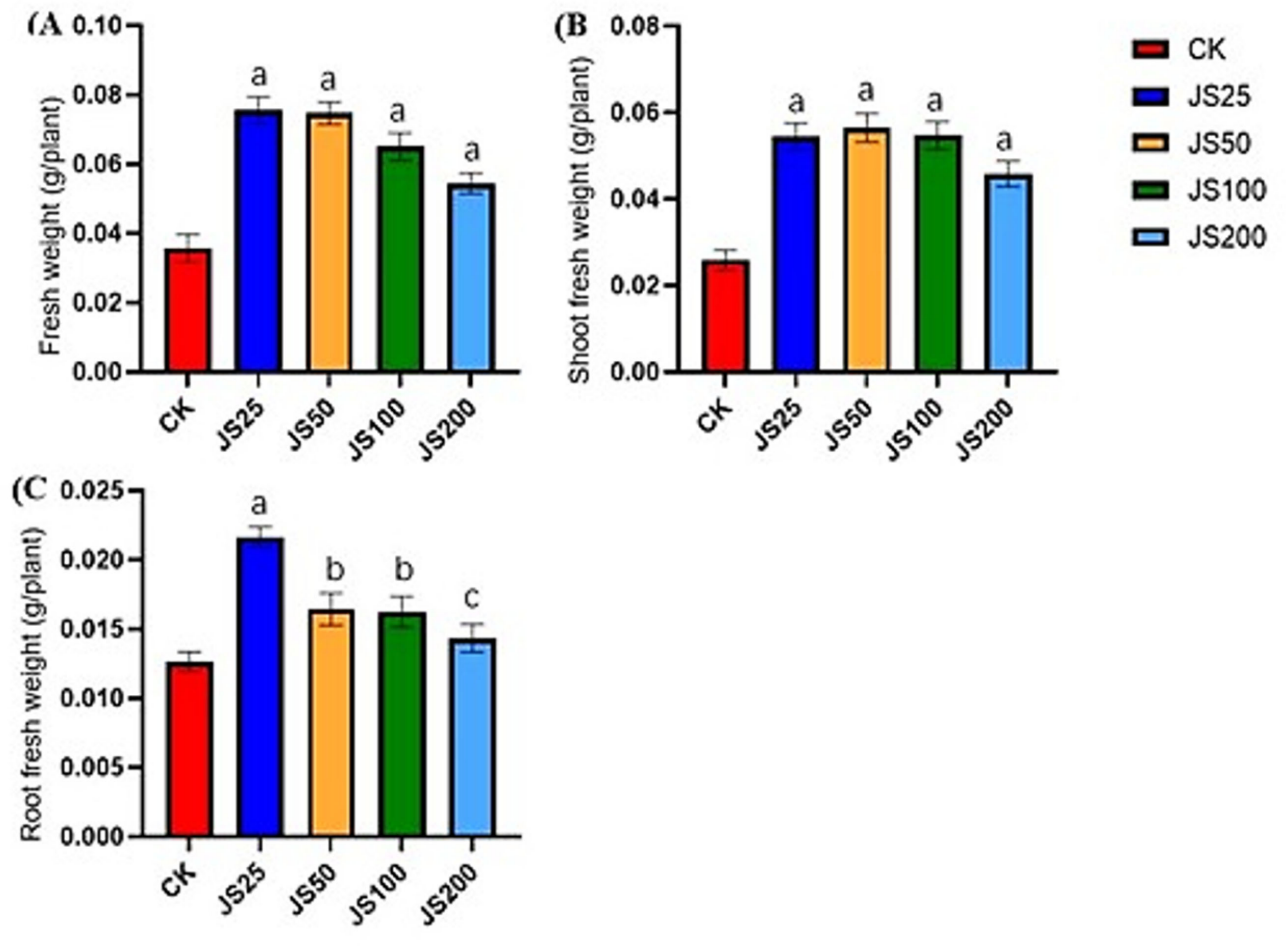
Effects of AFL on rice plant biomass under saline-alkali stress. **(A)** Total fresh weight; **(B)** Shoot fresh weight; **(C)** Root fresh weight. CK: control (deionized water); JS25, JS50, JS100, JS200: AFL dilutions of 1:25, 1:50, 1:100, and 1:200 (v/v), respectively. Data are presented as mean ± standard error (n = 3). ***P < 0.001, **P < 0.01 compared to CK.

Different concentrations of AFL had significant effects on the total fresh weight of rice (**Figure 3A**). The total fresh weight of JS25 treatment was the highest, reaching 0.0756 ± 0.0022 g/plant, which was significantly higher than that of the control (0.0357 ± 0.0023 g/plant) (P < 0.01). The total fresh weights of JS50 and JS100 treatments were 0.0747 ± 0.0019 g/plant and 0.0651 ± 0.0023 g/plant, respectively, which were also significantly higher that control (P< 0.001). The performance of each treatment was JS25 ~ JS50 > JS100> JS200> CK, and there was no significant difference between JS25 and JS50 treatments, indicating that the 1:25–150 dilution concentration had the best promotion effect on rice biomass accumulation.

##### 4.3.2.2 Shoot fresh weight

AFL treatment significantly increased shoot fresh weight in a concentration-dependent manner (**Figure 3B**). The shoot fresh weight of JS25 treatment reached 0.0738 ± 0.0018 g/plant, which was significantly higher than the control (0.0483 ± 0.0021 g/plant and 0.0587 ± 0.0020 g/plant, respectively, both significantly higher than the control (P< 0.01). JS200 treatment (0.0512 ± 0.0017 g/plant) also showed a significant increase compared to CK (P<0.01). The order was JS25 >JS50> JS100> JS200> CK.

##### 4.3.2.2 Root fresh weight

AFL treatment significantly promoted root fresh weight accumulation (**Figure 3C**). The highest root fresh weight was observed in JS25 treatment (0.0605 ± 0.0019 g/plant), followed by JS50 (0.0587 ± 0.0016 g/plant) and JS100 (0.0480 ± 0.0018 g/plant), all significantly higher than the control (0.0317 ± 0.0015) (P< 0.001). JS200 treatment (0.0447 ± 0.0014 g/plant) also showed a significant increase (P < 0.01). The treatment order was JS25 ~ JS50 > JS100> JS200>CK.

#### 4.3.3 Dry weight (total, shoot, and root)

**Figure 4:**
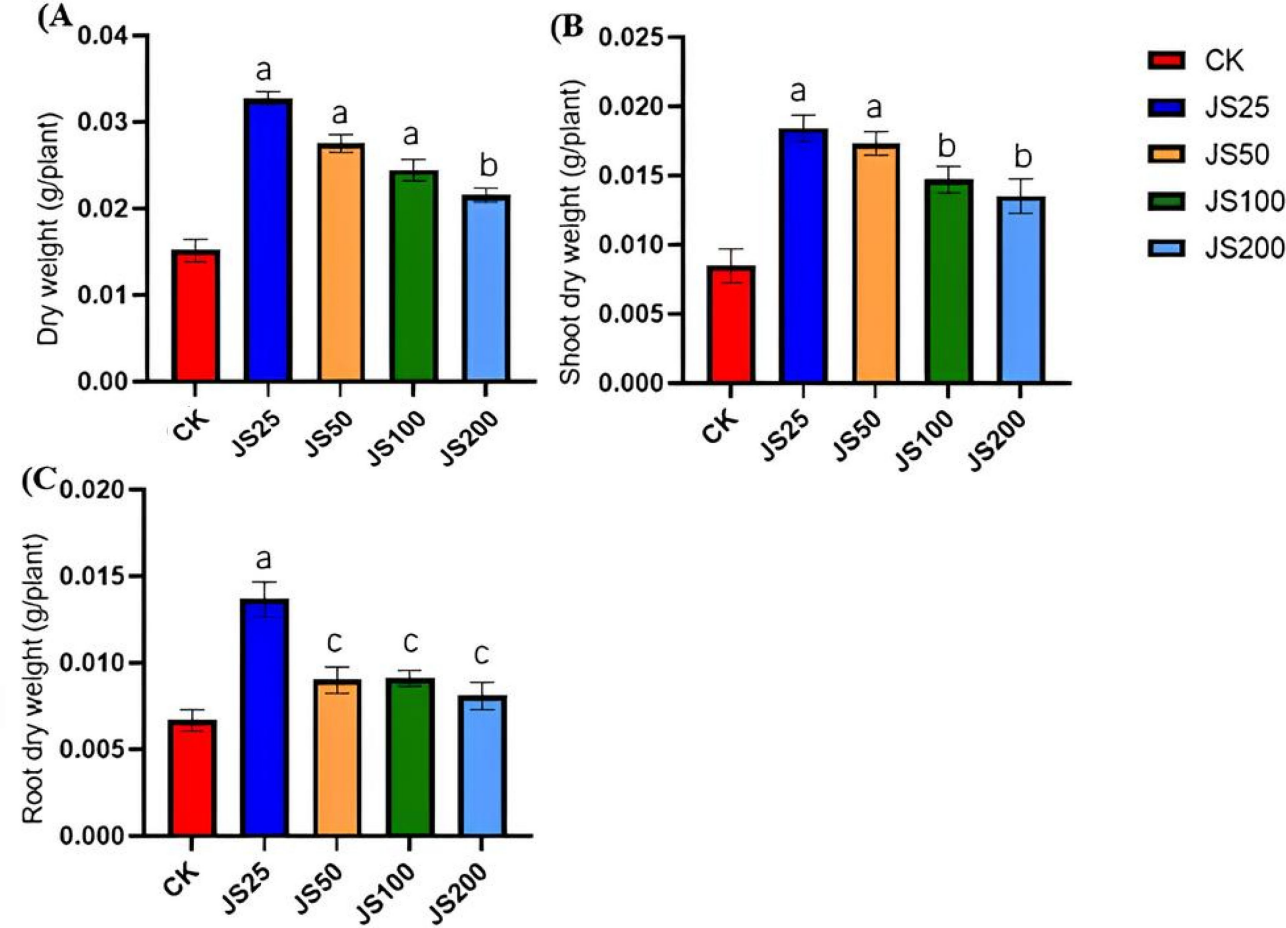
Effect of AFL on dry weight of rice plants. Note: **(A)** Dry weight; **(B)** Shoot dry weight; **(C)** Root dry wight. *** indicates extremely significant difference compared to the control group (CK) (P<0.001), ** indicates significant difference (P<0.01). CK: Control (deionized water); JS25, JS50, JS100, and JS200 represent 1:25, 1:50, 1:100, and 1:200 (v/v) dilutions of AFL, respectively. Data are mean ± standard error (n=3).

AFL treatment significantly increased dry matter accumulation in rice. The total dry weight of JS25 treatment was the highest, reaching 0.0327 ± 0.009 g/plant, which was significantly higher than that of the control (0.0152 ± 0.0013 g/plant) (P<0.001) (**Figure 4A**). The total dry weights of JS50 and JS100 treatments were 0.0275 ± 0.0010 g/plant and 0.0244 ± 0.0012 g/plant, respectively, which were also significantly higher than the control (P< 0.001). Shoot dry weight followed a similar pattern (**Figure 4B**, with JS25 (0.0198 ± 0.0007 g/plant) > JS50 (0.0165 ± 0.0006 g/plant) > JS100 (0.0142 ± 0.0008 g/plant) > JS200 (0.0125 ± 0.0005 g/plant) > CK (0.009 ± 0.0006 g/plant). Root dry weight showed the same trend, with JS25 (0.0129 ± 0.0004 g/plant) significantly higher than CK (0.0059 ± 0.0005 g/plant) (P < 0.001) (**Figure 4C**). These results demonstrate that AFL significantly promotes dry matter accumulation in rice under saline-alkali stress in a concentration-dependent manner.

#### 4.3.4 Rice culm diameter

**Figure 5:**
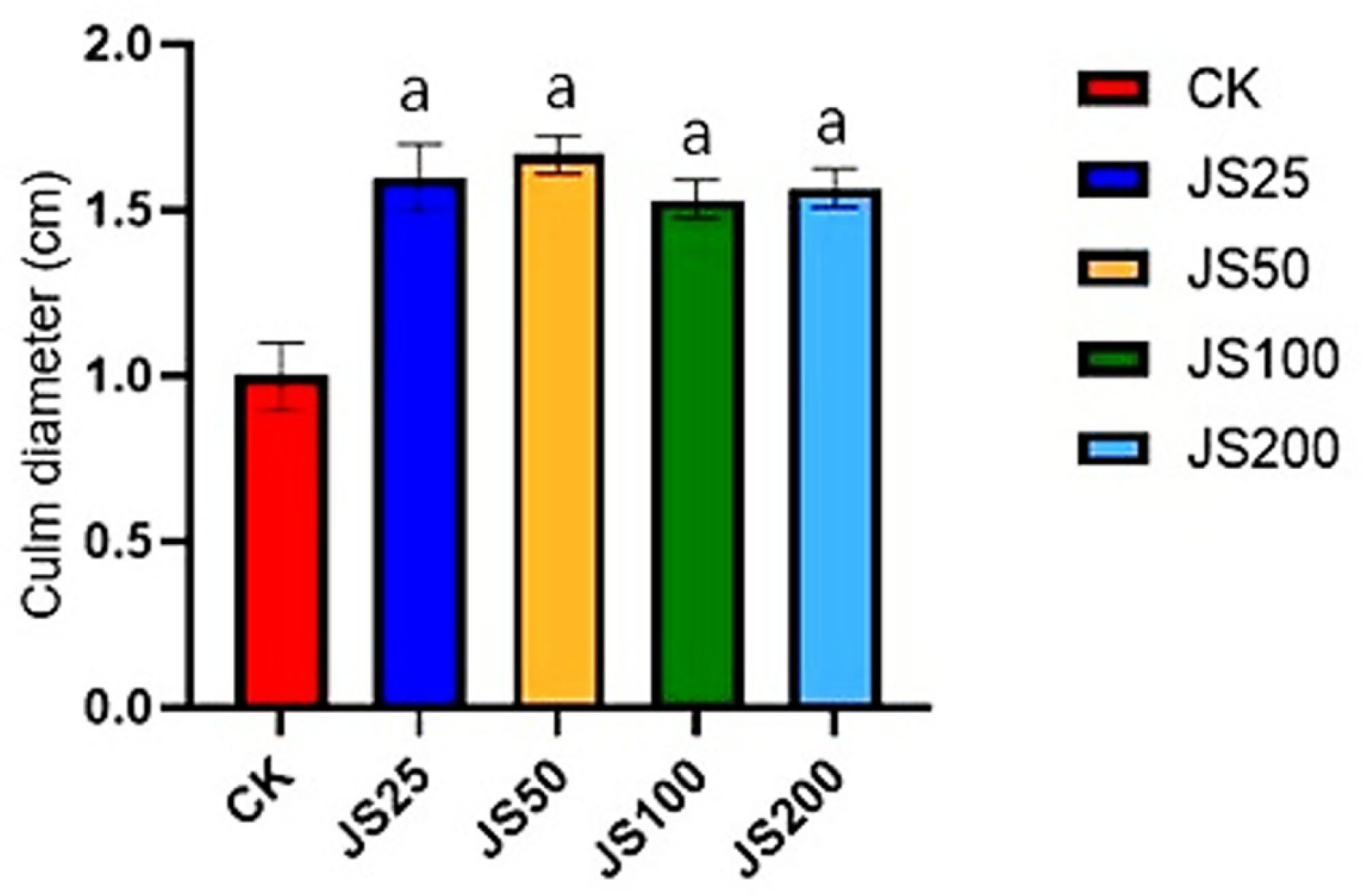
Effect of AFL on culm diameter of rice plants. Values with the similar letter (a) above the bars showing not significantly different. *** indicates an extremely significant difference compared to the control group (CK) (P<0.001), ** indicates significant difference (P<0.01). CK: Control (deionized water); JS25, JS50, JS100, and JS200 represent here, e.g., 1:25, 1:50, 1:100, and 1:200 (v/v) dilutions of AFL, respectively. Data are mean ± standard error (n=3).

Different concentrations of AFL had significant effects on the stem diameter of rice (**Figure 5**). The stem diameter of JS50 treatment was the largest, reaching 1.67 ± 0.06 cm, which was significantly higher than that of the control (1.00 0.10 cm, P < 0.001). The culm diameters of JS200, JS25 and JS100 treatments were 1.57 ± 0.06 cm, 1.60 ± 0.10 cm and 1.53 ± 0.06 cm, respectively, which were significantly higher than the control (P<0.001). There was no significant difference in stem diameter among the treatments, showing that JS50≈JS200≈JS25≈JS100>CK.

### 4.4 Root transcriptome reveals activation of stress-responsive pathways

In order to explore the physiological mechanism of AFL to alleviate saline-alkali stress, the key indexes such as chlorophyll content, proline content, antioxidant enzyme activity CAT, SOD and MDA content in rice leaves were determined. The results showed that different concentrations of AFL had significant effects on various physiological and biochemical indexes of rice leaves (**Table 3**).

**Table 3:** Effects of AFL on physiological and biochemical indexes of rice leaves.

Group	Total chlorophyll (mg/g)	Proline content (mg/100g)	CAT activity (U/g)	SOD activity (U/g)	MDA content (nmol/g)
CK	0.288 ± 0.003	13.456 ± 0.104	136.500 ± 1.725	138.688 ± 2.209	12.994 ± 0.154
JS200	0.42 ± 0.004***	10.233 ± 0.087***	189.567 ± 2.103***	167.234 ± 2.1956***	15.223 ± 0.32***
JS100	0.654 ± 0.002***	6.330 ± 0.097***	453.537 ± 1.514***	303.847 ± 2.177***	24.417 ± 0.147***
JS50	0.789 ± 0.003***	4.112 ± 0.072***	378.221 ± 2.345***	256.537 ± 2.345***	256.789 ± 2.011***
JS25	0.834 ± 0.002***	3.375 ± 0.060***	292.983 ± 2.540***	211.528 ± 2.362***	19.683 ± 0.189***
JS25	0.834 ± 0.002***	3.375 ± 0.060***	292.983 ± 2.540***	211.528 ± 2.362***	19.683 ± 0.189***

*P<0.001 compared to CK; data are mean ± SE (n=3) *.

#### 4.4.1 Chlorophyll content

The total chlorophyll content of JS25 treatment was the highest, reaching 0.834 0.002 mg/g, which was 2.90 times that of the control (0.288 0.003 mg/g), and the difference was extremely significant (P<0.001). The total chlorophyll content of JS100 treatment was 0.654 0.002 mg/g, which was also significantly higher than that of the control (P<0.001). The results showed that AFL could effectively promote the chlorophyll synthesis of rice and improve the photosynthetic potential.

#### 4.4.2 Proline content

Different from other indexes, the treatment of AFL significantly reduced the proline content in rice leaves. The proline content in the control group was as high as 13.456 ± 0.104 mg/100g g, while that in JS25 and JS100 treatments decreased to 3.375 ± 0.060 mg/100g g and 6.330 ± 0.097 mg/100g g, respectively, which were 74.9% and 53.0% lower than that in the control group (P < 0.001). Proline, as an important osmotic adjustment substance under plant stress, the decrease of its content shows that AFL effectively relieves the harm of saline-alkali stress to rice.

#### 4.4.3 Antioxidant enzyme activity

The activity of CAT and SOD in rice leaves was significantly improved by AFL treatment. In terms of CAT activity, JS100 treatment was the highest, reaching 453.537 1.514 U/g, which was 3.32 times that of the control (136.500 1.725 U/g). JS25 treatment was 292.983 2.540 U/g, which was 2.15 times that of the control (P<0.001). In SOD activity, JS100 treatment reached 303.847 2.177 U/g, which was 2.19 times that of the control (138.688 2.209 U/g). JS25 treatment was 211.528 2.362 U/g, which was 1.53 times that of the control (P<0.001).

#### 4.4.4 Analysis of phylum level

**Figure 6:**
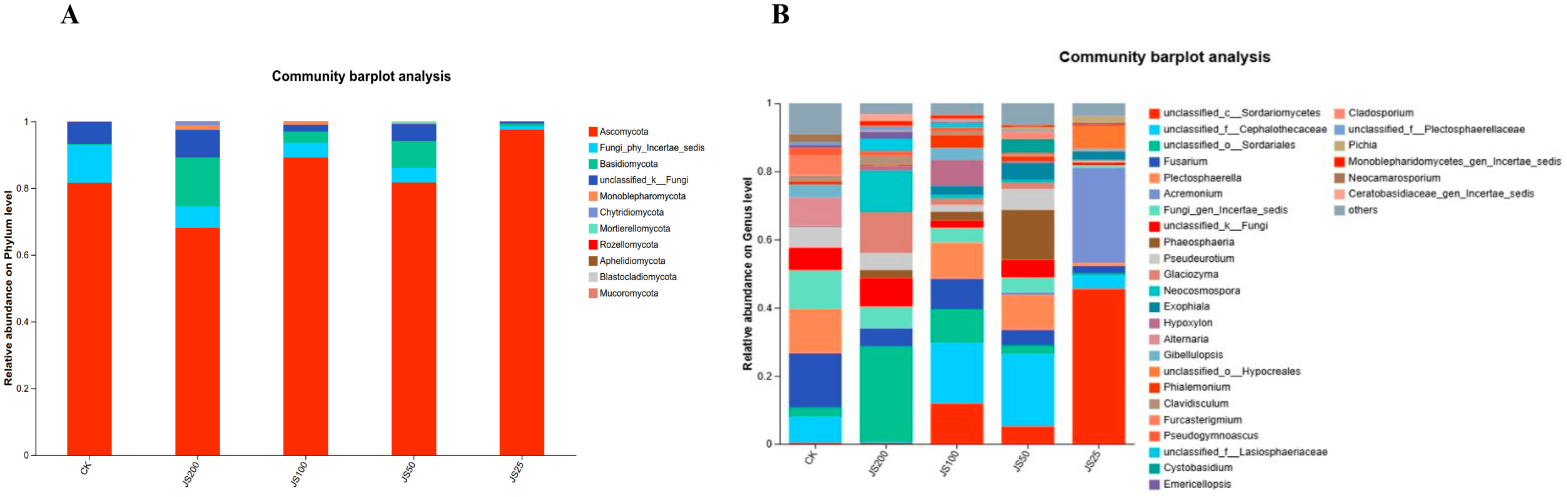
Composition of fungal community structure. Horizontal community analysis of phylum **(A)**; Genus level community analysis **(B)**.

The phylum level analysis showed (**Figure 6A**) that Ascomycota was the absolute dominant phylum in all treatment groups, and the relative abundance was over 80%. Other taxa, such as *Basidiomycota*, *Chytridiomycota* and *Mucoromycota*, are relatively low in abundance. There is little difference in the horizontal composition of phylum among different treatment groups, which indicates that the treatment of AFL mainly affects the fungal community structure at the genus level.

#### 4.4.5 Genus level analysis

Genus level community analysis showed (**Figure 6B**) that there were obvious differences in soil fungal community composition among different treatment groups. In CK group, *Emericellopsis*, *Cystobasidium*, *unclassified _ f _ lasiosphariaceae* are dominant. In JS200 group, the relative abundance of *Neocosmospora*, *Exophiala* and *Gibellulopsis* increased significantly. *Plectosphaerella* and unclassified_o_Hypocreales are the dominant genera in JS100 group. In JS50 and JS25 groups, *Plectosphaerella*, *Acremonium* and *Fusarium* were the dominant genera.

#### 4.4.6 Thermal map analysis of fungal community

**Figure 7:**
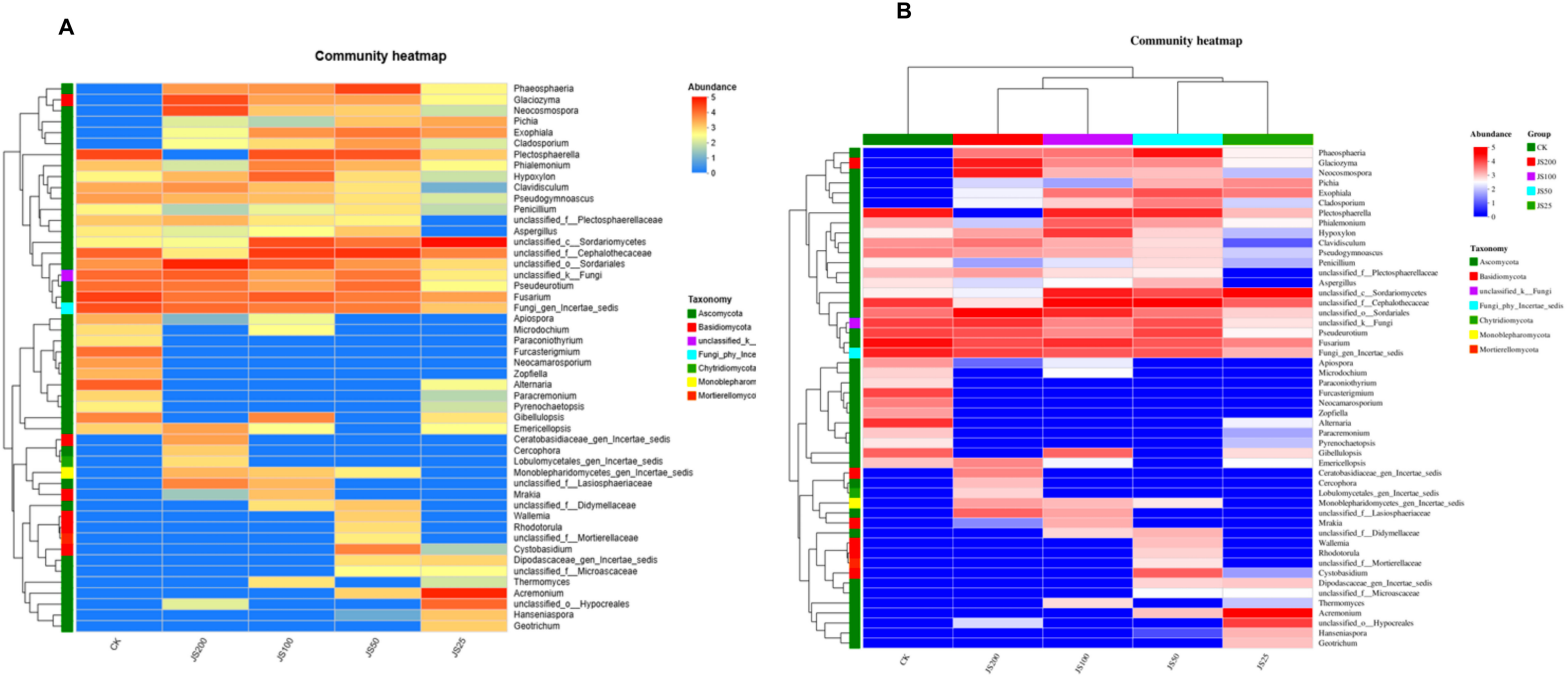
Thermal map analysis of fungal community.

**Figure 8:**
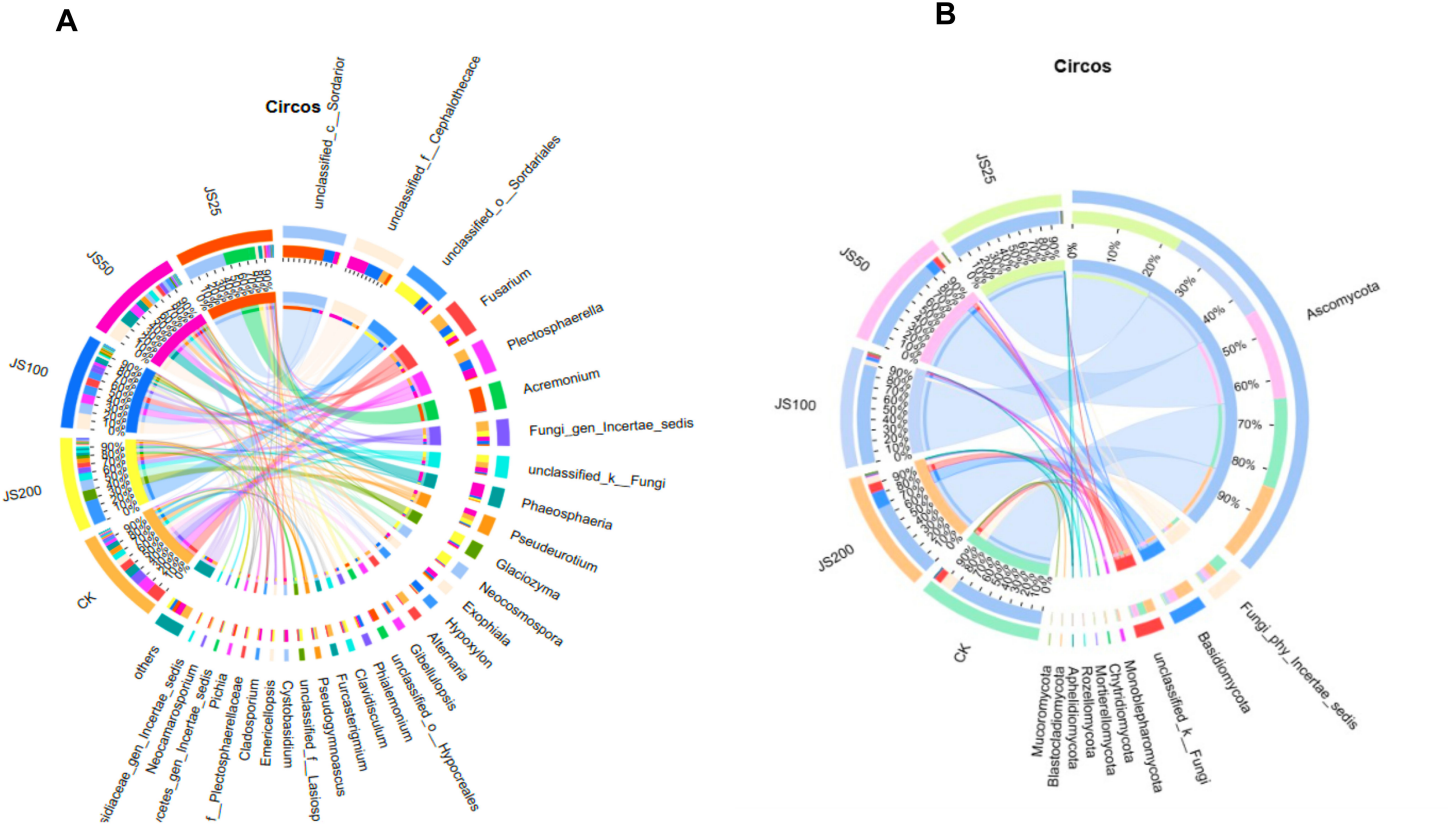
Circos diagram analysis of fungal community.

The analysis of community thermal map (**Figure 7**) shows that there are significant differences in the distribution of Top dominant species in different treatment groups. Cluster analysis showed that JS50 and JS25 were clustered into one group, JS100 and JS200 were clustered into another group, and CK group was clustered separately. *Plectosphaerella*, *Acremonium*, *Fusarium* and other genera have the highest abundance in JS50 and JS25 groups, showing obvious high abundance characteristics, while *Emericellopsis*, *Cystobasidium* and other genera have decreased abundance in these treatment groups (**Figures 8A**, **B**).

#### 4.4.7 Quality evaluation of transcriptome sequencing

**Figure 9:**
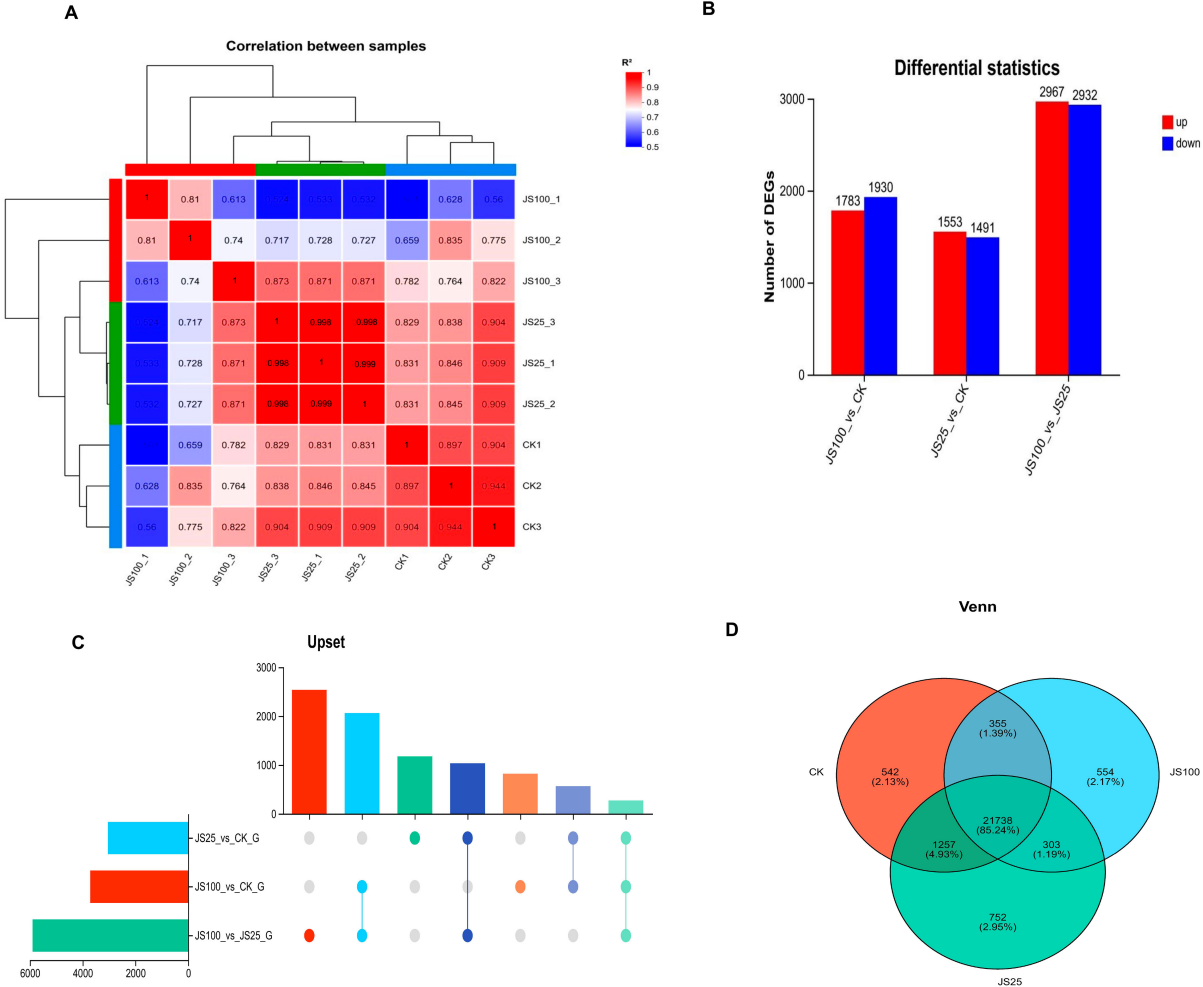
Transcriptome analysis of rice roots. **(A)** Sample correlation heat map; **(B)** statistics of differentially expressed genes; **(C)** Upset diagram analysis; **(D)** Venn diagram analysis.

Under the condition of saline-alkali soil cultivation, the correlation thermogram analysis between samples showed (**Figure 9A**) that the biological repetitions of each treatment group showed good consistency, indicating that the experimental design was reasonable and the data quality was reliable. The correlation between different treatment groups decreased obviously, which indicated that AFL significantly changed the gene expression pattern of rice under saline-alkali stress and had obvious stress-resistance regulation effect.

#### 4.4.8 Analysis of differentially expressed genes

The treatment of AFL significantly activated the expression of stress-resistant genes in rice (**Figure 9B**). Compared with saline-alkali stress control, JS100 high concentration treatment group activated 3713 differentially expressed genes, of which 1783 genes were up-regulated and 1930 genes were down-regulated. JS25 low concentration treatment group responded to 3044 differential genes, of which 1553 genes were up-regulated and 1491 genes were down-regulated. A direct comparison between JS100 and JS25 found 5899 differential genes, and the number of up-regulated genes (2967) was equivalent to that of down-regulated genes (2932), indicating that high concentration enzyme liquid phase could activate the expression of salt-alkali stress-resistant genes in rice more strongly for low concentration treatment.

The analysis of Upset diagram and Venn diagram (**Figures 9C, D**) showed that 272 differential genes (3.22%) shared by the three comparison groups may represent the core stress-resistant gene network of the effect of improving saline-alkali soil by apple fermentation liquid. The unique stress-resistance response genes treated with different concentrations of enzyme solution were: JS100 with 818 genes and JS25 with 1175 genes, indicating that the two concentrations had some different stress-resistance mechanisms. Compared with JS100_vs_JS25, there are 2535 unique differential genes with the largest number, which reflects the concentration-dependent stress-resistance regulation mode. The 564 genes co-regulated by the concentration of two kinds of fermentation liquids may be the key molecular targets for improving saline-alkali soil and enhancing the stress resistance of rice. These results reveal that AFL can alleviate salt-alkali stress in rice through multi-level and multi-channel gene regulation network.

## 5 Discussion

This study showed that AFL significantly promoted the growth and development of rice under saline-alkali stress. The plant height, stem length and root length of JS25 treatment increased by 70.7%, 111.4% and 72.9% respectively, and the dry matter accumulation increased by 115.1%. This result are consistent with the existing research, and the fruit and vegetable fermentation broth is rich in organic acids, amino acids, vitamins and plant growth regulators, which can effectively improve the saline-alkali soil environment and promote crop growth (Gao et al., 2024; Ma et al., 2025). However, we acknowledge an important limitation: because AFL have strong acidic (pH 3.17-3.24), can neutralize the high alkalinity of saline-alkali soil (pH 10.08), The observed growth promotion may be partially attributable to soil pH neutralization rather than solely to bioactive compounds. Without a pH-matched or acidified control treatment, we cannot definitively distinguish between these effects. This study was designed to evaluate the integrated effect of AFL as a complete amendment under realistic application conditions, and our interpretations reflect this multifactorial context.

The changes of physiological and biochemical indexes of leaves further provide insights into the responses associated to AFL treatment. In this study, it was found that the AFL treatment significantly increased the chlorophyll content of rice (JS25 treatment increased by 189.6%), which was closely related to the up-regulation of chlorophyll synthesis-related genes reported in the previous studies (Zhang et al., 2022; Li et al., 2024a). The increase of chlorophyll content directly enhances the photosynthetic efficiency and provides more photosynthetic products for plant growth. More importantly, the treatment with AFL significantly reduced proline content (JS25 treatment decreased by 74.9%), which is an important sign of reducing plant stress (Urmi et al., 2023; Riddech et al., 2025). Proline, as an important compatible solute for plants to cope with osmotic stress, important physiological indicator of reduce plant stress (Zulfiqar and Ashraf, 2023). The sharp decline in proline content observed in this study shows that AFL can effectively alleviate saline-alkali stress, so that plants can maintain normal osmotic balance without accumulating a lot of proline. At the same time, the activities of antioxidant enzyme systems (CAT and SOD) increased significantly (by 114.7%-232.2% and 52.5%-119.1% respectively), which enhanced the ability of plants to scavenge active oxygen and protected cells from oxidative damage (Kuk et al., 2003; Tavu and Redillas, 2025). These physiological responses together constitute the physiological basis for AFL may promote rice growth.

The content of MDA, a dependable biomarker for lipid peroxidation and oxidative membrane damage (Zhang, 2013), exhibited a concentration-dependent response to AFL treatment. Relative to the control (12.99 nmol/g), MDA levels rose to 15.22 nmol in JS200, 21.57 nmol/g in JS50, 19.68 nmol/g in JS25, and peaked at 24.42 nmol/g in JS100. Given that AFL increased the activity of antioxidant enzymes (CAT and SOD), this fist rise seemed contradictory. A hormesis-like response, however, can account for this pattern: low to moderate AFL concentrations (JS25, JS50, JS100) causes a brief oxidative burst that triggers antioxidant defense systems, whereas concurrent maximal CAT/SOD activities in JS100 may indicate optimal stress priming rather than uncontrollable damage. The slight decrease in MDA from JS100 to JS25 (24.42 to 19.68 nmol/g) indicates that oxidative priming may be suppressed by high AFL concentrations. Crucially, all AFL-treated plants exhibited much enhanced growth despite higher MDA, suggesting that generated oxidative signal is compatible with and may even aid in stress adaptation. The acknowledged function of regulated ROS signaling in plants stress response is consistent with study (Mittler et al., 2022).

An important finding of this study is that AFL has significantly changed the fungal community of saline-alkali soil, which providing a new perspective for understanding its soil improvement mechanism (Zhang et al., 2025). Consistent with studies examining fungal community shifts in response to soil amendments (Duan et al., 2024). The results of high-throughput sequencing showed that the relative abundance of fungal genera such as *Plectosphaerella*, *Acremonium*, *Fusarium* was significantly increased, while the abundance of *Emericellopsis*, *Cystobasidiu*m was decreased. We have interpreted these changes cautiously. While some, *Plectosphaerella* species have been associated with decomposition of organic matter and nitrogen cycling (Carlucci et al., 2012; Bi et al., 2023); Some species of *Acremonium* may promote plant growth (Khan et al., 2021), while, the genus *Fusarium* includes both beneficial and pathological species (Aoki et al., 2014) Without specie level identification or functional level evidence, we cannot classify these genera as “beneficial”. The observed shifts in fungal community structure may associated with rice growth through various potential mechanisms, including secretion of plant growth-promoting substances (Etesami, 2019; Devi et al., 2020; Kibret et al., 2024) or improved nutrient availability (Liu et al., 2016) or suppression of soil-borne pathogens (Ali et al., 2022). It is worth noting that the cluster analysis shows that the same fungal community structure of JS25 and JS50 treatment groups, which is quite consistent with its promotion effect on rice growth, suggesting that there is a close relationship between fungal community structure and plant growth performance. Similar shifts in fungal community composition in response to soil amendments have been documented in other crop systems (Guo et al., 2025). Importantly, because only ITS fungal sequence was performed, we cannot draw conclusions about bacterial community responses, and we have used fungal community rather than microbiome (O'Brien et al., 2005).

Transcriptome analysis revealed the mechanism of AFL regulating rice salt-alkali stress at molecular level. In this study, it was found that JS100 treatment showed 3713 differentially expressed genes, of which 1783 genes were up-regulated. These genes may be involved in many biological processes such as ion transport, osmotic adjustment, antioxidant defense, signal transduction and so on (Yang and Guo, 2018), Of particular concern are 272 core differential genes shared by the three comparison groups, which may represent the key molecular targets of improving saline-alkali soil associated with AFL treatment., These including genes encoding ion transporters, transcription factors, and antioxidant enzymes. Previous studies have shown that salt-tolerant plants maintain intracellular ion balance by up-regulating Na^+^/H^+^ antiporter gene (Almeida et al., 2017), and the activation of related genes in this study may be an important mechanism for rice adaptation to saline-alkali soil. In addition, there were 5899 different genes between JS100 and JS25 treatments, and the number of genes unique to the two concentrations are more (818 and 1175, respectively), which indicates that AFL with different concentrations act through some different molecular pathways and have a concentration-dependent regulation mode. We emphasize that these transcriptomic findings, are presented as exploratory and hypothesis-generating. This discovery provides a molecular basis for optimizing the application concentration of AFL and formulating precise improvement strategies.

This study demonstrates that AFL may be a suitable solution for saline-alkali soil, potentially addressing the problem through three complementary mechanism: possible direct chemical neuralization of alkalinity (inferred from AFL acidity, though soil pH was not measured post-treatment), organic matter-mediated fertility improvement, and observed activation of plant stress-defense pathways as evidenced by growth promotion, physiological changes, fungal community shifts, and transcriptomic reprogramming. The causal relationships among these observations remain to be established. From the theoretical prospective, this study integrated the multi-disciplinary technical means such as morphology, physiology, and molecular biology, constructed a relatively complete mechanism framework, and enriched the theoretical system of biological improvement of saline-alkali soil (Zhu et al., 2024). From the application level, AFL, as a resource utilization product of agricultural waste (fruit drop), has the advantages of easily available raw materials, simple preparation, low cost and environmental friendliness, which meets the requirements of green and sustainable agricultural development (Sharma et al., 2019). This study made it clear that 1:25-1:50 was the optimum concentration, which provided technical parameters for pot experiments. In addition, the core differential genes and key fungal groups found in this study can be used as molecular markers and fungal indicators to evaluate the improvement effect of saline-alkali land and screen efficient improvers (Etesami and Maheshwari, 2018). However, there are still some limitations in this study: (1) W have applied the pot experiments greenhouse conditions and there is differences between pot and the field environment, and field verification experiments are needed; (2) Only one crop of rice was studied, and the response characteristics of other main crops in saline-alkali soil (such as wheat, corn, cotton, etc.) need to be explored; (3) The effects of long-term application of AFL on soil quality and ecological environment need to be monitored continuously for many years. Future research should focus on field pilot tests, optimize application methods (ratio of basic application to topdressing, application frequency, etc.), explore the synergistic effect with other improvement measures (such as applying biochar and fungal agents, etc.), and establish a comprehensive evaluation system for saline-alkali soil improvement effect, laying the foundation for large-scale popularization and application.

## 6 Conclusions

This study demonstrated that, AFL application promotes rice growth in saline-alkali soil using pot experiment system under greenhouse conditions. The results showed that AFL significantly promote the growth and development of rice in saline-alkali soil, under dilution concentration of 1:25-1:50, promote the plant height, root length and dry matter accumulation increased by 70.7%, 72.9% and 115.1% respectively. Physiological analysis revealed that AFL treatment was associated with chlorophyll content, reducing proline accumulation and enhancing antioxidant enzyme activity (SOD and CAT), suggesting alleviation saline-alkali stress. Moreover, high-throughput ITS sequencing indicated that AFL application altered rhizosphere fungal community composition, with notable changes in the relative abundance of genera including *Plectosphaerella*, *Acremonium*, and *Fusarium*. Transcriptomic analysis identified 3713 differentially expressed genes in rice roots, following AFL treatment including 272 core genes potentially associated with stress adaptation pathways such as ion transport, antioxidant defense, and signal transduction. Overall, this study provides a multi-dimensional perspective on “soil fungal plant” interactions under AFL application and suggests its potential role in improving saline-alkali soil conditions. However, the direct contribution of specific fungal taxa to these effects requires further functional validation.

Further, the findings provide foundational framework for understanding multi-level responses associated with AFL application in saline-alkali soil and identifies specific targets for the future investigation. Future research should focus on validation studies, incorporate appropriate controls to unravel pH effects from bioactivity, examine bacterial community responses, validate candidate genes through qRT-PCR, and assess long term effects on soil health and crop productivity.

## Data Availability

The data presented in the study are deposited in the National Genomics Data Center (NGDC), accession numbers PRJCA070037, PRJCA068095.
